# Invasive leader cells: metastatic oncotarget

**DOI:** 10.18632/oncotarget.1870

**Published:** 2014-03-25

**Authors:** Kevin J. Cheung, Andrew J. Ewald

**Affiliations:** Departments of Cell Biology and Oncology, Center for Cell Dynamics, School of Medicine, Johns Hopkins University, Baltimore, MD, USA

Invasion into surrounding tissues is the first step of metastasis, and an understanding of the molecular basis of the invasive process may suggest novel strategies for anti-metastatic therapy. Breast tumors typically invade as adherent groups of cells, a process known as collective invasion. In vivo, collective invasion occurs within a complex tissue environment and involves interactions among the cancer cells, stromal cells, and the extracellular matrix (ECM). An additional challenge to understanding cancer invasion is that primary breast tumors contain diverse cancer cells with varied molecular phenotypes. It is difficult to determine the relative contribution of these subpopulations to invasion from analysis of histologic sections.

**Figure 1:**
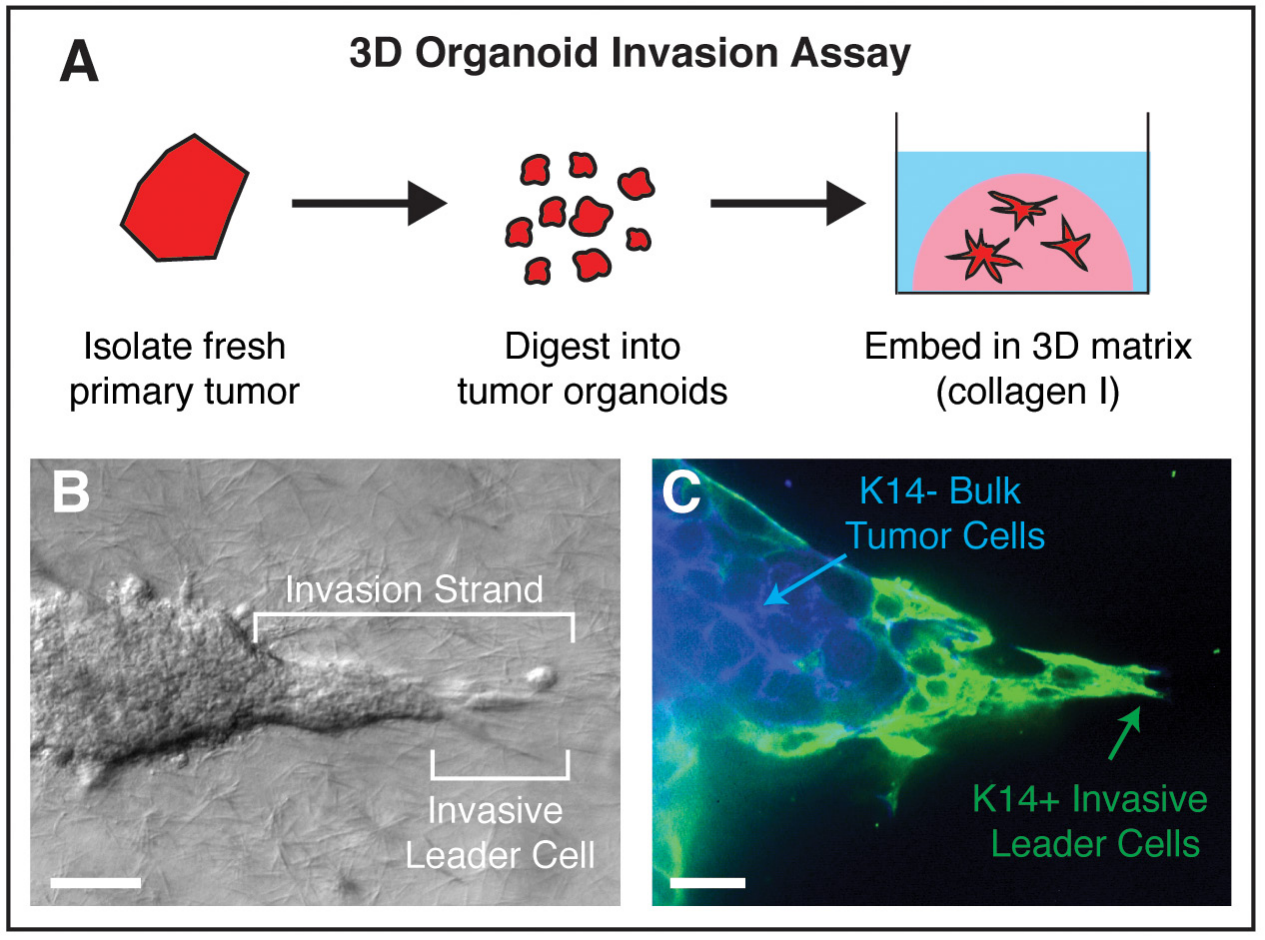
A. Tumor organoids are isolated from fresh primary tumors through a combination of mechanical disruption and enzymatic digestion. Tumor organoids are then embedded in 3D collagen I and become invasive. B. Organoids extend multiple invasive strands into the ECM, led by highly protrusive “invasive leader cells”. C. Invasive leaders cells are molecularly distinct from the bulk tumor cells and express markers of basal differentiation, such as keratin-14 (K14). Scale bar is 50 microns.

We sought to reduce this complexity and to identify the most invasive cancer cells in the primary tumor in an unbiased fashion [1]. To accomplish this goal, we developed a *three dimensional (3D) organoid invasion assay* (Figure 1A). Briefly, we use enzymes and mechanical disruption to isolate organoids from primary breast tumors from mouse models and human patients. Each organoid is composed of several hundred cancer cells and is embedded in an invasion promoting ECM [2] (collagen I). Tumor organoids invade collectively and the cells at the tips of invasion strands are protrusive and highly interactive with the ECM. We refer to the cells at the tips of these strands as invasive leader cells (Figure 1B).

We found that the molecular phenotype of invasive leader cells was distinct from the bulk tumor cells and conserved across breast cancer subtypes [1]. Leader cells expressed markers of basal epithelium, which are the cells in normal stratified tissues that interact with the ECM (Figure 1C). The basal cytokeratin, keratin-14 (K14), was expressed in ~90% of leader cells. Importantly, K14+ cells led collective invasion in multiple distinct mouse models and in diverse primary human breast tumors. We next used molecular biosensors to demonstrate that K14+ invasive leader cells were generated from non-invasive K14- bulk tumor cells. Importantly, knockdown of either K14 or p63, another leader cell marker, was sufficient to inhibit collective invasion.

Our data establish three important concepts. First, we have demonstrated that the invasive behavior of the primary tumor is determined by a subset of specialized cancer cells^1^. Second, our data suggest a common molecular biology underlying breast cancer metastasis, because K14+ cells led collective invasion in 3D organoid invasion assays from patients with different breast cancer subtypes. In support of this concept, multiple groups have observed that K14 expression in breast tumors correlates with poor patient outcomes, independent of breast cancer subtype [3-5]. Third, since bulk tumor cells can convert into K14+ invasive leaders, we hypothesize that inhibition of the basal molecular program could control progression in patients with metastatic disease.

Our 3D organoid invasion assay can also be utilized to identify invasive leader cells in other epithelial cancers. Because basal cells are found in many stratified epithelial tissues, the basal invasive program we identified may be utilized across diverse cancers. In support of this concept, basal cytokeratins are associated with invasion in both lung adenocarcinoma and hepatocellular carcinoma [6,7].

It is now critical to develop the translational framework to leverage our knowledge of invasive leader cells to improve patient outcomes. We are specifically interested to determine whether the abundance of invasive leader cells in the primary tumor will provide independent information about the prognosis of individual patients. Our invasion assays also provide an ideal platform to identify anti-invasive therapeutic compounds. Now that we have the invasive leader cell in our sights, we are working to defeat this new adversary.
